# Primary pulmonary synovial sarcoma with delayed diagnosis in a 69-year-old man: A case report

**DOI:** 10.1097/MD.0000000000036620

**Published:** 2023-12-22

**Authors:** Chan Seop Kim, Hye Soo Cho, Ok Jun Lee, Su Yeon Ahn, Jin Young Yoo

**Affiliations:** a Department of Radiology, Chungbuk National University Hospital, Cheongju, Korea; b Department of Radiology, Seoul National University Hospital, Seoul, Korea; c Department of Pathology, Chungbuk National University College of Medicine, Cheongju, Korea; d Department of Radiology, Konkuk University Medical Center, Konkuk University School of Medicine, Seoul, Korea; e Department of Radiology, Chungbuk National University College of Medicine and Hospital, Cheongju, Korea.

**Keywords:** coarse intratumoral calcifications, delayed diagnosis, primary pulmonary synovial sarcoma, radiologic features

## Abstract

**Rationale:**

Primary pulmonary synovial sarcoma is a rare malignant pulmonary tumor accompanied by calcifications in approximately 15% of cases. These calcifications usually have a fine, stippled appearance; coarse shapes have seldom been reported. Moreover, the presence of coarse calcifications often suggests benign tumors, which vastly differ in treatment. We present a rare case of primary pulmonary sarcoma with coarse intratumoral calcifications, the diagnosis of which was delayed because of its radiologic appearance.

**Patient concerns:**

A computed tomography (CT) scan of a 69-year-old man with right upper quadrant (RUQ) pain revealed an incidental mass at the base of the right lower lobe, the margin of which was not well described with respect to the liver, and intratumoral coarse calcification was noted. Initially, the lesion was believed to be hepatic, and magnetic resonance imaging (MRI) was performed. Based on its imaging features, the mass was thought to be a pulmonary lesion, and a preliminary diagnosis of a benign lesion, such as a hamartoma or granuloma, was made. Four months after the initial CT scan, the patient’s RUQ pain had aggravated; however, no change in the mass was observed on follow-up CT.

**Diagnosis:**

The final diagnosis was primary pulmonary sarcoma, proven by surgical biopsy.

**Interventions:**

Wedge resection of the right lower lobe was performed, and the patient received adjuvant chemotherapy.

**Outcomes:**

The patient’s RUQ pain improved, and no recurrence or metastasis has been reported to date.

**Lessons:**

This case describes a rare presentation of a primary pulmonary synovial sarcoma with coarse intratumoral calcifications and the MRI features of the lesion. Intratumoral coarse calcifications often suggest benign lesions, such as hamartomas or post-inflammatory granulomas; however, as malignant lesions cannot be completely excluded, other radiologic and clinical features should be considered carefully. Focal areas of enhancement and eccentric calcification distribution might suggest malignant lesions such as primary pulmonary synovial sarcoma. Furthermore, despite not being used routinely, MRI scans might be helpful because advanced MRI techniques, such as diffusion-weighted imaging, can help distinguish malignant lesions from benign lesions. If the clinical course of a patient suggests malignancy, a more aggressive biopsy strategy should be considered.

## 1. Introduction

Synovial sarcoma, which accounts for 5% to 10% of all soft tissue sarcomas, is a malignant soft-tissue tumor that usually arises near the articular structure. However, “synovial sarcoma” is a misnomer because this type of sarcoma originates from primitive mesenchymal cells rather than from the intra-articular synovium. It can occur anywhere in the body, with the most common site being the extremities. Most pulmonary synovial sarcomas are metastases from extrathoracic synovial sarcomas, and primary sarcomas are rare, accounting for <0.5% of all pulmonary malignancies.^[1]^

A synovial sarcoma is accompanied by calcifications in approximately 30% of cases,^[2]^ but in primary pulmonary synovial sarcoma, its frequency is reported to be approximately 15%.^[3]^ These calcifications mostly appear as fine, stippled calcifications; coarse calcifications are seldom reported.^[4]^ In terms of evaluating intratumoral calcifications on computed tomography (CT), stippled and punctate calcifications suggest a tumor’s malignant potential, while the presence of intratumoral coarse, popcorn-like, or laminated calcifications suggests benign tumors such as hamartoma or inflammatory granuloma. Thus, diagnosis of primary pulmonary synovial sarcoma can be delayed because of a lack of early symptoms and indolent growth.^[1,2,4]^

We herein report a case of primary pulmonary synovial sarcoma presenting with large coarse calcifications, which was initially believed to be a benign lesion but was later diagnosed as primary pulmonary synovial sarcoma via surgical biopsy.

## 2. Case presentation

This study was approved by the Institutional Review Board of Chungbuk National University Hospital (CBNUH 2023-09-024) and was CARE-compliant. Written informed consent was obtained from the patient for the publication of this report and any accompanying images.

**Figure 1.**
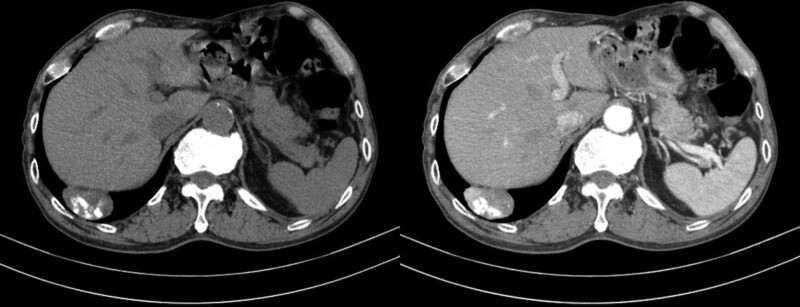
Initial computed tomography (CT) scan of the mass. Axial pre- and post-contrast mediastinal setting CT images (slice thickness, 3 mm) show a 45-mm, well-defined mass at the right lower lobe, abutting the diaphragm. The nodule has well-defined coarse calcifications and heterogeneous enhancement.

**Figure 2.**
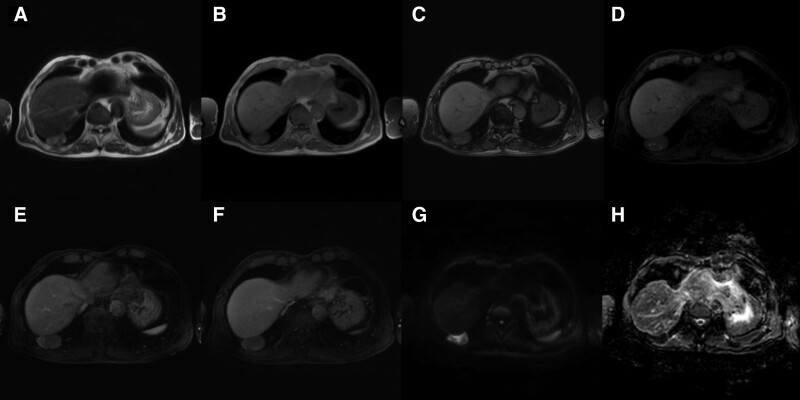
Magnetic resonance imaging (MRI) scan during workup. (A) T2-weighted MRI (slice thickness, 5 mm) shows a 45-mm lesion with a heterogeneous, intermediate-intensity signal with internal foci of high signal intensity. In-phase (B) and out-of-phase (C) T1-weighted images show no signal drop in the out-of-phase image, suggesting the absence of a microscopic fat component. Pre-contrast (D), arterial-phase post-contrast (E), and portal-phase post-contrast (F) T1-weighted images show heterogeneous enhancement consistent with previously scanned pre- and post-contrast CT images. (G) b800 diffusion-weighted MRI. (H) The apparent diffusion coefficient (ADC) map shows a mean ADC value of 972, suggesting diffusion restriction within the mass.

A 69-year-old man with right upper quadrant pain was referred to our hepatology department. A CT scan performed outside the facility revealed a 4.5-cm circumscribed mass at the base of the right lower lobe, abutting the right diaphragm, with heterogeneous enhancement and intratumoral eccentric coarse calcification (Fig. 1). No mediastinal lymphadenopathy was observed. The mass was at an acute angle with the diaphragm, suggesting a pulmonary parenchymal origin rather than a hepatic, diaphragmatic, or pleural origin. However, some of its margins were not well described with respect to the liver and diaphragm, and magnetic resonance imaging (MRI) of the liver was performed. On MRI, its margins with the liver and diaphragm were well described, and the possibility of it being a hepatic mass was excluded. The mass showed heterogeneous but overall intermediate signal intensity on T1- and T2-weighted imaging and foci of high signal intensity within the mass on T2-weighted imaging. On post-contrast T1-weighted imaging, the mass showed heterogeneous enhancement, consistent with the previous CT image. There was no evidence of microscopic fat on the in-phase and out-of-phase T1-weighted images. Notably, on diffusion-weighted imaging, the tumor showed remarkable diffusion restriction, with an apparent diffusion coefficient value of 972 (Fig. 2). Laboratory studies were unremarkable other than serum carcinoembryonic antigen, which was minimally increased (5.13 ng/mL). However, this finding was also considered unremarkable because of the minimal increase. The patient’s medical or familial history of cancer was unremarkable. Considering these radiological features and laboratory results, the tumor was considered likely to be a benign lesion, such as a hamartoma or a post-inflammatory granuloma. However, as some portions of the tumor showed enhancement and the calcifications were eccentric, the possibility of malignancy could not be excluded. The patient was transferred to the thoracic surgery department, and the surgeon, based on the report of the radiologic department, opted to perform a follow-up CT scan after 6 months.

**Figure 3.**
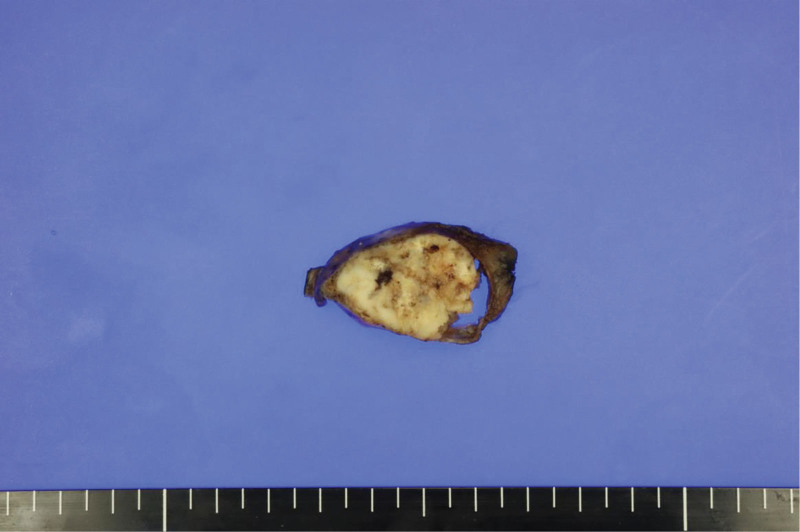
Macroscopic findings. The cut surface of the resected well-circumscribed mass shows a whitish-gray tumor with calcifications, measuring 5.5 cm in the largest diameter. The tumor was confined to the lung parenchyma, and its pleural surface was smooth.

**Figure 4.**
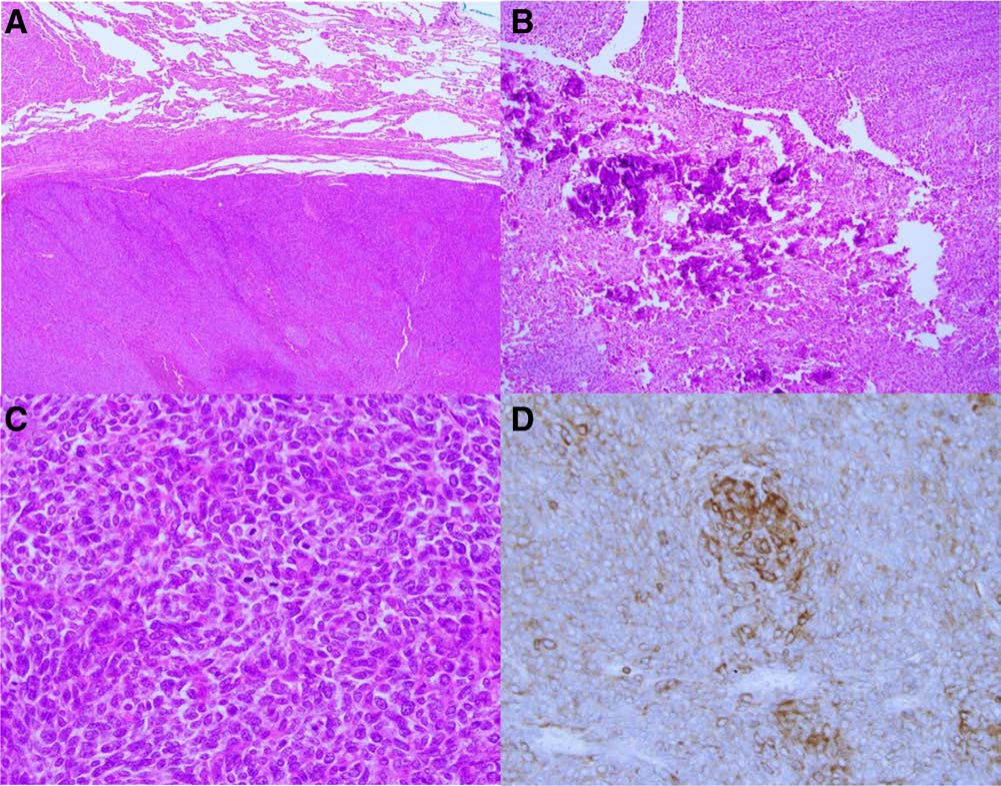
Histological findings after surgical biopsy. (A–C) Hematoxylin and eosin-stained microscopic images show a well-defined margin between the mass and normal lung parenchyma (original magnification × 40) (A), internal foci of calcifications (original magnification × 100) (B), and densely populated short spindle cells and collagen deposits between tumor cells (original magnification × 400) (C). (D) Immunochemistry stain for BCL2 was positive. These findings are consistent with synovial sarcoma.

Four months after the initial CT scan, the patient revisited the outpatient thoracic surgery clinic, complaining of aggravated pain in the right upper quadrant. Follow-up CT did not show any significant interval change in the mass, and wedge resection of the right lower lobe was performed for surgical biopsy. Grossly, the cut surface of the resected, well-circumscribed mass showed a whitish-gray tumor with calcifications measuring 5.5 cm in diameter. The tumor was confined to the lung parenchyma, and its pleural surface was smooth (Fig. 3). Histopathologically, the tumor was composed of densely populated short spindle cells with internal calcification foci and collagen deposits. Immunohistochemically, the tumor cells tested positive for CD99 and BCL2. Based on these findings, the tumor was diagnosed as a monophasic synovial sarcoma (Fig. 4). As pathologic findings cannot differentiate metastatic pulmonary synovial sarcoma from extrathoracic synovial sarcoma or primary pulmonary synovial sarcoma, a whole-body fluorodeoxyglucose–positron emission tomography (FDG-PET)/CT scan was performed, which showed no evidence of extrathoracic lesions; thus, the diagnosis of primary pulmonary synovial sarcoma was confirmed. After surgery, the patient’s symptoms improved, and he received adjuvant chemotherapy.

## 3. Discussion

Primary pulmonary synovial sarcoma is an aggressive tumor with a 5-year survival rate of 50%. In most cases, it lacks early symptoms. When symptomatic, it presents as a large, heterogeneous thoracic mass with pleuritic chest pain, cough, dyspnea, hemoptysis, or pleural effusion. On CT, it appears as a circumscribed mass with heterogeneous enhancement and occasional calcifications. These radiologic features are nonspecific, and differential diagnosis from other tumors such as intrathoracic sarcoma other than synovial sarcoma, solitary fibrous tumor of the pleura, thymic neoplasm, or lung cancer is needed. Even if synovial sarcoma is confirmed by surgical biopsy, it is not immunohistopathologically distinguishable from metastatic and primary synovial sarcomas. Furthermore, the lungs are the most common sites of metastasis from extrathoracic synovial sarcoma. Therefore, the diagnosis of primary pulmonary synovial sarcoma requires systematic investigation to exclude other possible diagnoses.^[1,5]^ Primary pulmonary synovial sarcoma rarely accompanies lymphadenopathy^[5]^; thus, if a large circumscribed mass is present in young adults without lymphadenopathy, not only lung cancer but also primary pulmonary synovial sarcoma should be considered. Whole-body FDG-PET/CT may be useful for excluding metastatic synovial sarcomas because synovial sarcomas tend to show avid FDG uptake.^[5,6]^

Synovial sarcomas are classified into 3 histologic subtypes: biphasic, monophasic, and poorly differentiated. Among these, biphasic synovial sarcoma is the most common type^[1]^; however, the monophasic type is much more common in the respiratory system.^[5]^ The histopathologic features in our case showed densely populated short spindle cells with areas of collagen fibers and focal calcifications consistent with synovial sarcoma.

When a pulmonary nodule contains intranodal calcification, it is known as a central location, and a large coarse morphology such as popcorn-like or laminated calcification can virtually exclude malignancy; however, eccentric, punctate, or amorphous calcification suggests the potential for malignancy. A wide variety of entities can appear as nodules with calcification, from benign nodules such as hamartomas or granulomas to malignant conditions such as primary lung cancer and primary intrathoracic or metastatic sarcomas.^[7]^ Considering the broad spectrum of these entities, the possibility of malignancy should be evaluated when the calcification pattern does not exactly fit the benign pattern but the location of the nodule correlates with the patient’s symptoms.

Our case appeared as a circumscribed, heterogeneous, enhancing mass with intratumoral calcification, which is known as a primary pulmonary synovial sarcoma.^[1]^ However, calcifications in primary pulmonary sarcomas tend to be punctate or amorphous,^[2,5]^ which did not occur in our case, as calcifications appeared well-defined and coarse. Because of the nonspecific radiologic features of primary pulmonary synovial sarcoma and the discrepancy with its known calcification pattern, it could be misdiagnosed as a benign condition, such as hamartoma or post-inflammatory granuloma. However, on account of the diffusion-weighted MRI scan, we decided not to exclude the possibility of malignancy, which helped in an accurate diagnosis. Although MRI is not routinely used for the diagnosis of lung nodules, in this case, it was performed incidentally, and the tumor showed diffusion restriction, as previously reported.^[8]^ However, the generalizability of these MRI findings in primary pulmonary sarcoma is limited because of the small sample size. Further studies are needed to verify the MRI findings of primary pulmonary synovial sarcoma.

## 4. Conclusion

The case presented here shows a radiologic picture of a rare primary pulmonary sarcoma with large coarse calcifications. Because of its slow-growing nature and calcification, it can be easily overlooked as a benign lesion. Active clinical and radiologic surveillance could help in the accurate diagnosis, and the use of nonroutine diagnostic modalities, such as diffusion-weighted MRI, can also be helpful in ambiguous lesions.

## Author contributions

**Conceptualization:** Jin Young Yoo.

**Data curation:** Chan Seop Kim, Ok Jun Lee.

**Investigation:** Chan Seop Kim, Ok Jun Lee.

**Visualization:** Chan Seop Kim, Jin Young Yoo.

**Writing – original draft:** Chan Seop Kim, Jin Young Yoo.

**Writing – review & editing:** Chan Seop Kim, Hye Soo Cho, Su Yeon Ahn, Jin Young Yoo.
